# Upregulation of desmoglein 2 and its clinical value in lung adenocarcinoma: a comprehensive analysis by multiple bioinformatics methods

**DOI:** 10.7717/peerj.8420

**Published:** 2020-02-13

**Authors:** Ruiying Sun, Chao Ma, Wei Wang, Shuanying Yang

**Affiliations:** 1 Department of Respiratory and Critical Care Medicine, the Second Affiliated Hospital of Xi’an Jiaotong University, Xi’an, Shaanxi, China; 2 Department of Anesthesiology, Xi’an Children Hospital, Xi’an, Shaanxi, China

**Keywords:** DSG2, Lung adenocarcinoma, Prognosis, TCGA

## Abstract

**Background:**

Desmoglein-2 (DSG2), a desmosomal adhesion molecule, is found to be closely related to tumorigenesis in recent years. However, the clinical value of DSG2 in lung adenocarcinoma remains unclear.

**Methods:**

Real-time reverse transcription-quantitative polymerase chain reaction (qRT-PCR) was utilized to detect the expression of DSG2 in 40 paired lung adenocarcinoma tissues and corresponding non-cancerous tissues. Data from The Cancer Genome Atlas (TCGA) and Oncomine datasets were also downloaded and analyzed. The correlation between DSG2 and clinicopathological features was investigated. The expression of DSG2 protein by immunohistochemical was also detected from tissue microarray and the Human Protein Atlas database. Integrated meta-analysis combining the three sources (qRT-PCR data, TCGA data and Oncomine datasets) was performed to evaluate the clinical value of DSG2. Univariate and multivariate Cox regression analyses were used to explore the prognostic value of DSG2. Then, co-expressed genes were calculated by Pearson correlation analysis. Gene Ontology (GO) enrichment analysis and Kyoto Encyclopedia of Genes and Genomes (KEGG) analysis were used to investigate the underlying molecular mechanism. The expression level in lung adenocarcinoma and prognostic significance of the top ten co-expressed genes were searched from Gene Expression Profiling Interactive Analysis (GEPIA) online database.

**Results:**

DSG2 was highly expressed in lung adenocarcinoma tissues based on qRT-PCR, TCGA and Oncomine datasets. The protein expression of DSG2 was also higher in lung adenocarcinoma. According to qRT-PCR and TCGA, high DSG2 expression was positively associated with tumor size (*p* = 0.027, *p* = 0.001), lymph node metastasis (*p* = 0.014, *p* < 0.001) and TNM stage (*p* = 0.023, *p* < 0.001). The combined standard mean difference values of DSG2 expression based on the three sources were 1.30 (95% confidence interval (CI): 1.08–1.52) using random effect model. The sensitivity and specificity were 0.73 (95% CI [0.69–0.76]) and 0.96 (95% CI [0.89–0.98]). The area under the curve based on summarized receiver operating characteristic (SROC) curve was 0.79 (95% CI [0.75–0.82]). Survival analysis revealed that high DSG2 expression was associated with a short overall survival (hazard ratio [HR] = 1.638; 95% CI [1.214–2.209], *p* = 0.001) and poor progression-free survival (HR = 1.475; 95% CI [1.102–1.974], *p* < 0.001). A total of 215 co-expressed genes were identified. According to GO and KEGG analyses, these co-expressed genes may be involved in “cell division”, “cytosol”, “ATP binding” and “cell cycle”. Based on GEPIA database, seven of the top ten co-expressed genes were highly expressed in lung adenocarcinoma (DSC2, SLC2A1, ARNTL2, ERO1L, ECT2, ANLN and LAMC2). High expression of these genes had shorter overall survival.

**Conclusions:**

The expression of DSG2 is related to the tumor size, lymph node metastasis and TNM stage. Also, DSG2 predicts poor prognosis in lung adenocarcinoma.

## Introduction

Lung cancer is considered to be the leading cause of cancer death in both men and women worldwide. Lung adenocarcinoma (LUAD), accounts for approximately 40% of all lung cancers, is the most common subtype and leads to more than 500,000 deaths each year (Ferlay et al., 2010). For all stages combined, the survival of lung cancer is about 18%. However, most lung cancer is typically diagnosed at a distant stage which causes the 5-year survival rates is 5% (Siegel, Miller & Jemal, 2018). Thus, it is important to identify the potential diagnostic and prognostic markers for patients with LUAD.

Desmosomal cadherins family is one major subfamily of cadherins which comprised two different cadherins—desmogleins and desmocollins (Lowndes et al., 2014). Desmogleins consist of DSG1, DSG2, DSG3 and DSG4. DSG2 is expressed in all desmosome-bearing tissues and some epithelial tissues, such as cardiac muscle, skin and intestinal epithelium (Schafer, Stumpp & Franke, 1996; Ungewiss et al., 2018). As a member of cadherins, DSG2 is an important regulator of growth and survival signaling pathways, cell proliferation, apoptosis, migration and invasion and oncogenesis (Brennan et al., 2007; Cooper et al., 2018; Nava et al., 2007). Loss of DSG2 function in humans is directly linked to arrhythmogenic right ventricular cardiomyopathy (ARVC), an autosomal recessive disease underpinned by myocyte apoptosis and fibrous degeneration of the myocardium (Pilichou et al., 2009). Studies showed that DSG2 was aberrantly expressed in many tumors, such as gastric cancer (Biedermann et al., 2005), skin squamous cell carcinomas (Kurzen, Munzing & Hartschuh, 2003) and melanoma (Tan et al., 2016). Trojan *et al* revealed that increased expression of DSG2 was found in the metastatic prostate cancer cell line compared to its non-metastatic syngeneic precursor cell (Trojan et al., 2005).

Moreover, DSG2 has an impact on the occurrence and development of many tumors and is closely related with diagnosis and prognosis of cancers. For instance, Davie *et al* reported that DSG2 was a putative tumor suppressor and could reduce cell aggregation, invasion, and motility in human breast cancer cells (Davies et al., 1997). Another study revealed that reduced level of DSG2 was a significant independent marker of poor clinical outcome (Barber et al., 2014). To date, few studies have reported that DSG2 might be an early diagnostic and independent prognostic marker for lung adenocarcinoma (Cai et al., 2017; Saaber et al., 2015). The molecular mechanisms of DSG2 in lung adenocarcinoma remain largely elusive and require further investigation.

In this study, the clinical value of DSG2 was evaluated. Firstly, a total of 40 paired lung adenocarcinoma tissues and corresponding non-cancerous tissues were used to detect the expression of DSG2. Data from The Cancer Genome Atlas (TCGA) and Oncomine datasets were also downloaded and analyzed. The expression level of DSG2 protein by immunohistochemical was also detected by the tissue microarray and the Human Protein Atlas (HPA) database. The correlation between DSG2 and clinicopathological features was investigated. Secondly, integrated meta-analysis was performed to evaluate the clinical value of DSG2. Survival analysis was evaluated by univariate and multivariate Cox regression analyses. Thirdly, co-expressed genes were calculated by Pearson correlation analysis. Gene Ontology (GO) enrichment analysis and Kyoto Encyclopedia of Genes and Genomes (KEGG) pathway analysis were used to investigate the underlying molecular mechanism. The expression between LUAD and normal lung tissues and prognostic significance of the top ten co-expressed genes were searched from Gene Expression Profiling Interactive Analysis (GEPIA) online database.

## Material and Methods

### Patients and tissues

Forty paired lung adenocarcinoma tissues and non-cancer tissues (>five cm adjacent to the tumor) were collected from patients who underwent pulmonary surgery in the department of thoracic surgery in our hospital from March 2016 to June 2019. All patients had no history of other cancers and underwent no other prior treatment including chemotherapy, radiotherapy or targeted therapy before surgery. This study was approved by the Ethics Committee of the Second Affiliated Hospital of Xi’an Jiaotong University (2016036). Written informed consent was obtained from all participants.

### Tissue microarray (TMA) and immunohistochemistry

Tissue microarray (HLugA150CS03) containing 75 paired lung adenocarcinoma tissues and adjacent non-tumor tissues was purchased from Shanghai Outdo Biotech (Shanghai, China). Then immunohistochemistry (IHC) assay was performed. Tissue sections were treated with EDTA buffer under high pressure at high temperature to retrieve antigen. Sections were incubated with primary antibody DSG2 (1:250, ab150372, Abcam, USA) at 4 ^∘^C overnight. Biotinylated anti-IgG was added and incubated at room temperature for 1 hour. The slides were stained with DAB and counterstained with haematoxylin after incubating with streptomycin-HRP for 30 minutes. Samples were visualized using diaminobenzidine system and hematoxylin re-dying, and analyzed under microscope (OLYMPUS CX41, Japan).

Tissue microarrays were scored independently by two pathologists who were blinded to the clinicopathologic features and outcomes of the patients. The staining was analyzed under low magnification (40×) and high magnification (200×). The intensity of staining was divided into four grades (intensity scores): negative (0), weak (1), moderate (2) and strong (3). Immunoreactivity was divided into five grades (percentage scores) according to the percentage of stained cells: no staining (0), 1%–25% (1), 26%–50% (2), 51%–75% (3) and >75% (4). Then, a final histological overall score was calculated by the multiplication of the two values. An overall score of 0–12 was calculated and graded as low (score < 7) or high (score ≥ 7).

### Qrt-pcr

Fast1000 (Xfyangbio, China) was used to extract the total RNA from all the specimens according to the manufacturer’s protocol. After the reverse transcription, the TB Green^®^ Premix Ex Taq™ II (Takara Biotechnology Co., Ltd.) was utilized to detect the gene amplification and qRT-PCR was performed on the CFX96 Touch™ Real-Time PCR Detection System (Bio-rad) in standard mode for 40 cycles. The primers were as follows: ACTIN forward, 5′-TGGCACCCAGCACAATGAA-3′  and reverse, 5′-CTAAGTCATAGTCCGCCTAGAAGCA-3′; DSG2 forward, 5′- ATGACGGCTAG GAACACCAC-3′  and reverse, 5′-GGGTCAGTTTGTGGCTGACT-3′(Vite et al., 2013). All experiments were conducted in triplicate. The difference of DSG2 expression between cancer tissues and non-cancer tissues was calculated using the formula 2^−Δ*Cq*^.

### Data from online databases

RNA-Seq data of DSG2 in LUAD were downloaded from TCGA (https://portal.gdc.cancer.gov/). After repeated values were deleted, LUAD consisted of 513 lung adenocarcinoma tissues and 59 lung normal tissues. Among them, there were 57 paired tissues. The original expression data were displayed in fragments per kilobase million (FPKM). All the data were transformed into TPM (transcripts per million reads) and normalized by log_2_(TPM+1) for the following analyses. Clinical information including age, gender, tumor-node-metastasis (TNM) stage, smoking status were also obtained. The original data from other studies were downloaded from Oncomine database (http://www.oncomine.org/). Also, the expression level of DSG2 protein was detected from the Human Protein Atlas (http://www.proteinatlas.org/) (Ponten, Jirstrom & Uhlen, 2008).

### Integrated meta-analysis

A comprehensive meta-analysis was performed to determine the combined expression value of DSG2 in LUAD groups and non-cancer groups by combining the three sources (qRT-PCR data, TCGA data and Oncomine datasets). I-squared test was used to evaluate heterogeneity. When I^2^<50%, a fixed-effects model was used, while I^2^>50%, the random-effects model was selected (Higgins et al., 2003). Standard mean difference (SMD) with a 95% confidential interval (CI) was used to assess the effect sizes of these pooled data. A summary receiver operating characteristic (SROC) curve was constructed to describe the diagnostic ability of DSG2 in LUAD. In addition, Begg’s test was carried out to confirm whether publication bias existed in the studies.

### Co-expressed genes and pathways identification and analysis

In order to find out potential mechanism, Pearson test was used to identify co-expressed genes. The cutoff value was set as —R—>0.4 and *P* < 0.05. The interaction between DSG2 and co-expressed genes were processed by the Cytoscape 3.7.1 software to generate a visual image. GO enrichment and KEGG pathway analysis of these co-expressed genes were performed by Database for Annotation, Visualization and Integrated Discovery (DAVID) online database (https://david.ncifcrf.gov/) (Huang da, Sherman & Lempicki, 2009). *P* < 0.05 and false discovery rate (FDR) <0.05 were considered to have significance significantly. GEPIA (http://gepia.cancer-pku.cn/index.html) contains the RNA sequencing expression data of 9,736 tumors and 8,587 normal cases from TCGA and the Genotype-Tissue Expression (GTEx) projects (Tang et al., 2017). In the present study, GEPIA was used to compare the expression levels of the co-expressed genes between lung adenocarcinoma tissues and normal tissues and investigate the prognostic significance.

### Statistical analysis

All data were analyzed using R software (version 3.5.1) and GraphPad Prism 8. Meta-analysis was analyzed using STATA 15. The student’s *t*-test was used to compare the differential expression level of DSG2 between LUAD tissues and adjacent normal lung tissues. The associations between the level of DSG2 and clinicopathological parameters were evaluated using Mann-Whitney *U* test or Kruskal–Wallis *H* test. The chi-squared test was utilized to determine whether there was a difference in the distribution of DSG2 samples among the different categories in the TMA. The prognostic value was evaluated by Cox regression analyses and Kaplan–Meier. Multivariate Cox analysis was used to compare the impact of DSG2 expression on survival along with other clinical traits, the median DSG2 expression was regarded as the cut-off value. The associations between DSG2 expression and co-expressed genes were determined by Pearson correlation coefficient. All *p* value <0.05 was considered statistically significant.

## Results

### Clinical value of DSG2 expression in lung adenocarcinoma

**Figure 1:**
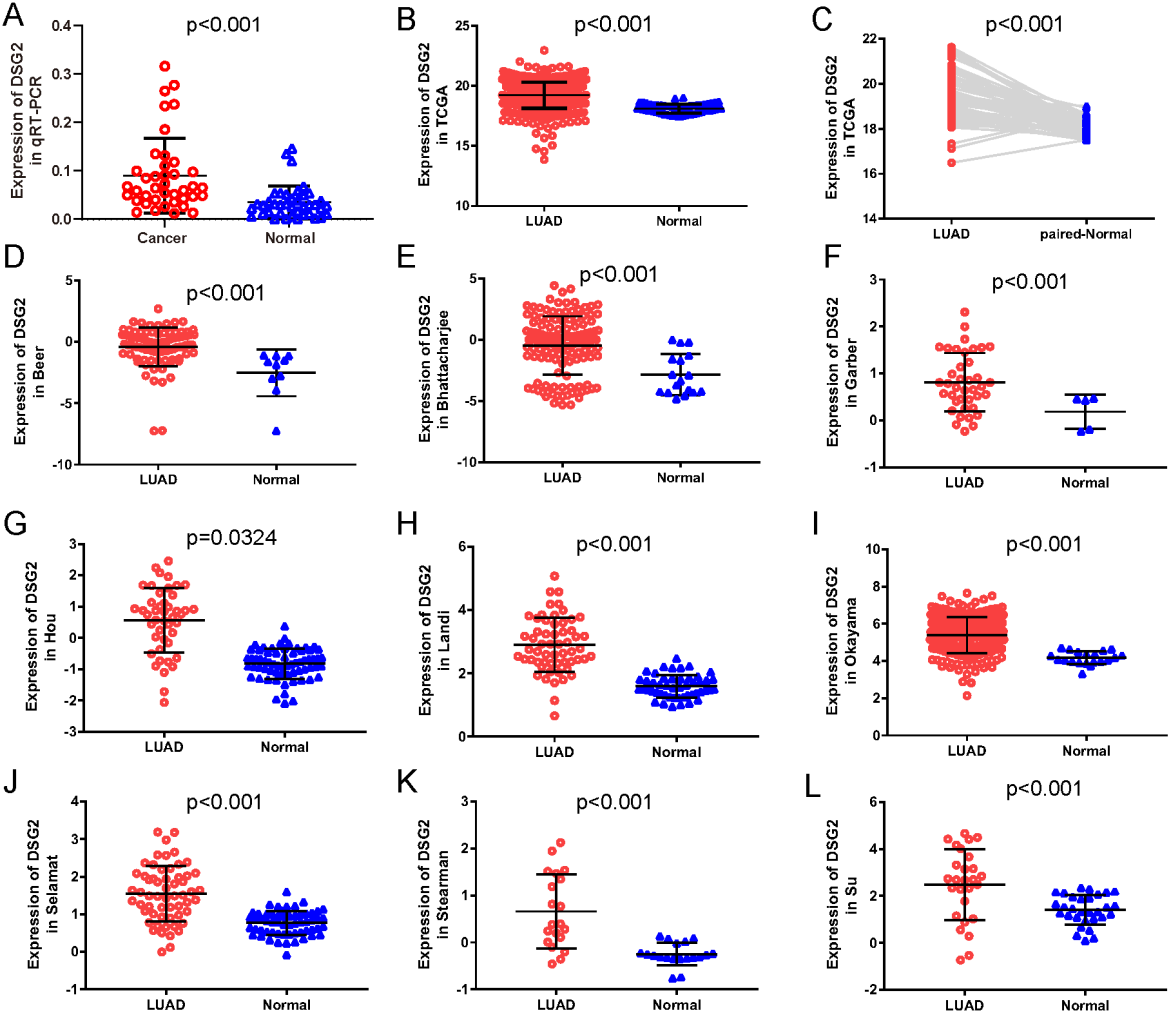
The expression of DSG2 in lung cancer tissues and normal lung tissues. (A) Forty paired lung adenocarcinoma and non-cancerous lung tissue was detected by qRT-PCR. (B) A total of 513 LUAD and 59 normal lung tissues from TCGA. (C) Fifty-seven paired LUAD and normal lung tissues from TCGA. (D–L) Nine studies from Oncomine datasets.

In the current study, a total of 40 paired lung adenocarcinoma samples were utilized to determine the expression of DSG2 by qRT-PCR. As shown in Fig. 1A, DSG2 was significantly over-expressed in lung adenocarcinoma than corresponding non-tumorous normal tissues (*p* < 0.001). To further identify the importance of DSG2 in the lung adenocarcinoma progression, the correlation between DSG2 and clinicopathological features was analyzed. The expression level of DSG2 in patients with early TNM tumor stage (I–II) was lower than patients with advanced TNM stage (III–IV) (0.063 ± 0.040 *vs* 0.128 ± 0.098, *p* = 0.023). For tumors greater than three cm in size, a significant increase in DSG2 expression was observed (0.109 ± 0.087 *vs* 0.052 ± 0.027, *p* = 0.027). Patients with lymph node metastasis exhibited higher DSG2 expression than patients without lymph node metastasis (0.113 ± 0.087 *vs* 0.053 ± 0.034, *p* = 0.014). No significant association was found between DSG2 expression and other clinicopathological characteristics, including age, gender and smoking history (Table 1).

**Table 1:** Correlation between the expression of DSG2 and the clinicopathological characteristics of the lung adenocarcinoma patients.

Clinicopathological features		*n*	Mean ± SD	*p*-value
Age (years)	<60 ≥60	23 17	0.089 ± 0.074 0.092 ± 0.084	0.845
Gender	Male Female	18 22	0.080 ± 0.058 0.099 ± 0.090	0.973
Smoking history	No Yes	24 16	0.099 ± 0.086 0.077 ± 0.058	0.759
TNM tumor stage	I+II III+IV	23 17	0.063 ± 0.040 0.128 ± 0.098	0.023^*^
Tumor size (cm)	≤3 >3	13 27	0.052 ± 0.027 0.109 ± 0.087	0.027^*^
Lymph node metastasis	No Yes	15 25	0.053 ± 0.034 0.113 ± 0.087	0.014^*^

**Notes.**

* *p* < 0.05; TNM, tumor-node-metastasis; SD, standard deviation.

To further verify our results, data from other studies including TCGA data and Oncomine datasets were used. Original data consisted of 513 LUAD samples and 59 normal lung samples with clinicopathological information were downloaded from TCGA. Among them, there were 57 paired LUAD samples. As shown in Fig. 1B, increased expression of DSG2 was observed in cancer tissues compared to normal lung tissues (*p* < 0.001). The analysis of the paired LUAD samples exhibited a consistently higher expression level in lung cancer (Fig. 1C). In addition, the associations between DSG2 expression and clinicopathological parameters were shown in Table 2. A higher expression of DSG2 is significantly correlated with a higher T stage (*p* = 0.001), N stage (*p* < 0.001) and TNM stage (*p* < 0.001). Also, the expression of DSG2 was closely related with gender (*p* = 0.017). With regard to age, smoking history and M stage, no statistical significance was observed. Nine datasets about LUAD were downloaded from Oncomine online database. In all the studies, DSG2 has a significant higher expression in cancer tissues than lung normal tissues (Figs. 1D–1L).

**Table 2:** Relationship between the expression of DSG2 and clinicopathological features in LUAD based on the TCGA.

Clinicopathological features		*n*	Mean ± SD	*p*-value
Age (years)	<60 ≥60	136 358	6.441 ± 1.233 6.360 ± 1.084	0.419
Gender	Male Female	237 276	6.488 ± 1.180 6.283 ± 1.099	0.017^*^
Smoking history	No Yes	12 499	6.648 ± 0.987 6.374 ± 1.145	0.439
T	T1 T2 T3 T4	168 276 47 19	6.156 ± 1.041 6.436 ± 1.251 6.589 ± 0.776 6.898 ± 0.697	0.001^**^
N	N0 N1 N2	330 95 74	6.236 ± 1.180 6.538 ± 1.016 6.823 ± 1.047	<0.001^**^
M	M0 M1	344 25	6.423 ± 1.109 6.473 ± 0.804	0.786
TNM tumor Stage	I II III IV	274 121 84 26	6.173 ± 1.193 6.522 ± 1.096 6.798 ± 1.010 6.500 ± 0.801	<0.001^**^


**Notes.**

* *p* < 0.05.

** *p* < 0.01.

T tumor N node M metastasis TNM , tumor-node-metastasis SD standard deviation

**Figure 2:**
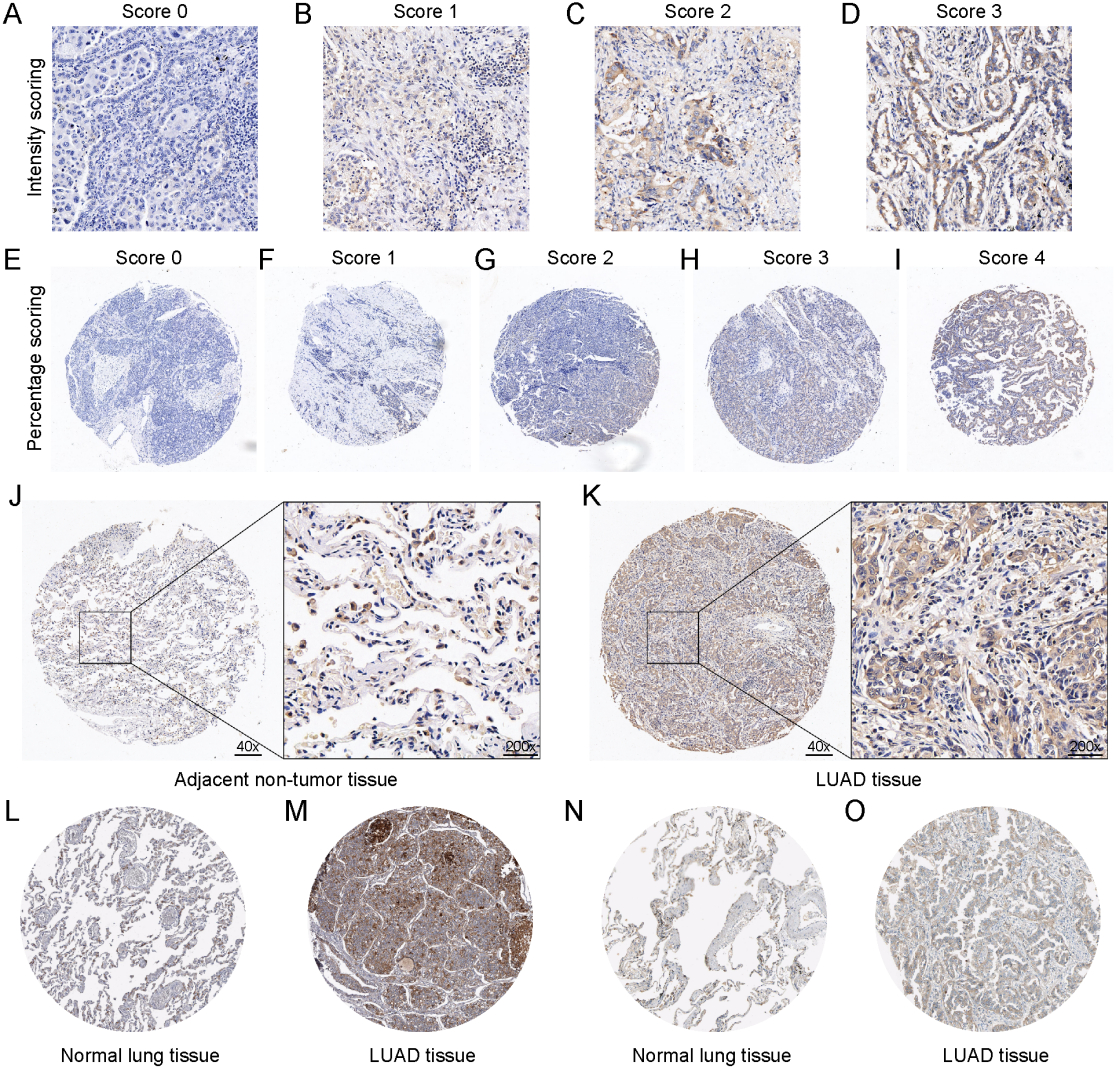
The protein expression of DSG2 in LUAD tissues and non-tumor tissues. The scoring system of the tissue microarray was displayed. A final histological overall score was calculated by the multiplication of the intensity score (A–D) and percentage score (E–I). Representative images of IHC staining of DSG2 in 60 adjacent non-tumor tissues (J) and 62 LUAD tissues (K). DSG2 protein expression was searched from the Human Protein Atlas database. CAB025122 antibody was used in normal lung tissue (L) and LUAD tissue (M). HPA004896 antibody was used in normal lung tissue (N) and LUAD tissue (O).

In addition, tissue microarray was used to verify the expression level of DSG2. The system scoring was shown in Figs. 2A–2I. Immunohistochemistry staining analysis showed higher expression of DSG2 in LUAD tissues compared with the adjacent non-tumor tissues (Figs. 2J–2K, *p* < 0.001). Higher DSG2 expression was positively associated lymph node metastasis (*p* = 0.040). However, there was no correlation between DSG2 expression and age, tumor size or TNM stage (Table 3, *p* >  0.05). Higher DSG2 expression was also observed in the male group than the female group (*p* < 0.001). DSG2 was located in the cytoplasm or membranous. Moreover, immunostaining evidence from the Human Protein Atlas database supported the upregulation of DSG2 in lung adenocarcinoma tissues. Two different antibodies (HPA004896 and CAB025122) were utilized to detect the expression of DSG2 in 9 lung adenocarcinoma tissues and 6 normal lung tissues. By antibody CAB025122, 3 were high staining and 2 were medium. By using antibody HPA004896, of total 4 lung adenocarcinoma tissues, 3 were high staining and 1 was medium. However, staining in normal lung tissues were both low no matter which antibody was used (Figs. 2L–2O).

**Table 3:** Association between the DSG2 expression in LUAD tissues and the patients’ clinical characteristics based on the tissue microarray.

Clinicopathological features		*n*	DSG2 expression		*p*-value
			Low	High	
Tissues	Non-tumor tissues LUAD tissues	60 62	60 33	0 29	<0.000^**^
Age (years)	<60 ≥60	27 35	14 19	13 16	0.849
Gender	Male Female	32 29	10 22	22 7	<0.001^**^
T	T1 T2-3	10 52	8 25	2 27	0.064
N	N0 N1-3	31 29	21 12	10 17	0.040^*^
TNM tumor Stage	I-II III-IV	37 25	23 10	14 15	0.086

**Notes.**

* *p* < 0.05.

** *p* < 0.01.

T tumor N node TNM tumor-node-metastasis

### Integrated meta-analysis

**Figure 3:**
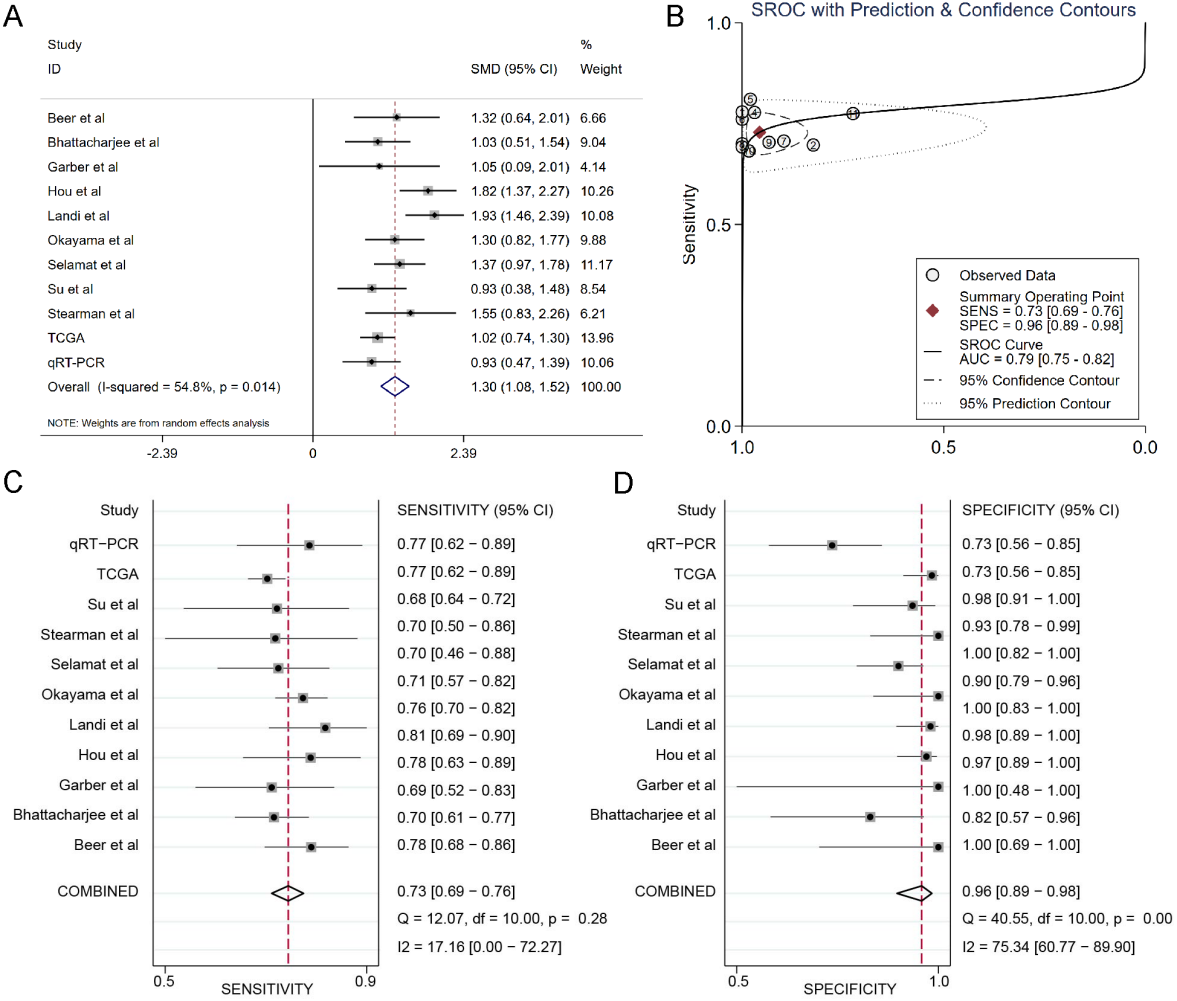
Integrated meta-analysis based on qRT-PCR, TCGA and 9 Oncomine datasets. (A) Forest plot of the DSG2 expression. (B) SROC of DSG2 in the LUAD within the total 11 datasets included. Sensitivity (C) and specificity (D) analysis of DSG2. The black squares represent the sensitivity /specificity value of each dataset. The horizontal lines indicate the 95% confidence intervals of each study. The diamonds represent the effect size.

The results of the integrated meta-analysis contained TCGA data, Oncomine datasets and qRT-PCR data. As shown in Fig. 3A, the pooled effect sizes from forest plots indicated that DSG2 was significantly highly expressed in lung adenocarcinoma than in non-cancer tissues (SMD=1.30, 95% CI [1.08–1.52], *p* = 0.014) using the random effect model (I^2^=54.8%). Area under curve (AUC) for the diagnostic value of DSG2 was 0.79 (95% CI [0.75–0.82]) (Fig. 3B). The pooled sensitivity and specificity were 0.73 (95% CI [0.69–0.76]) (Fig. 3C) and 0.96 (95% CI [0.89–0.98]) (Fig. 3D), respectively. Apart from that, Begg’s test revealed that no publication bias existed among all the studies analyzed (*p* = 0.615).

### Survival analysis

**Figure 4:**
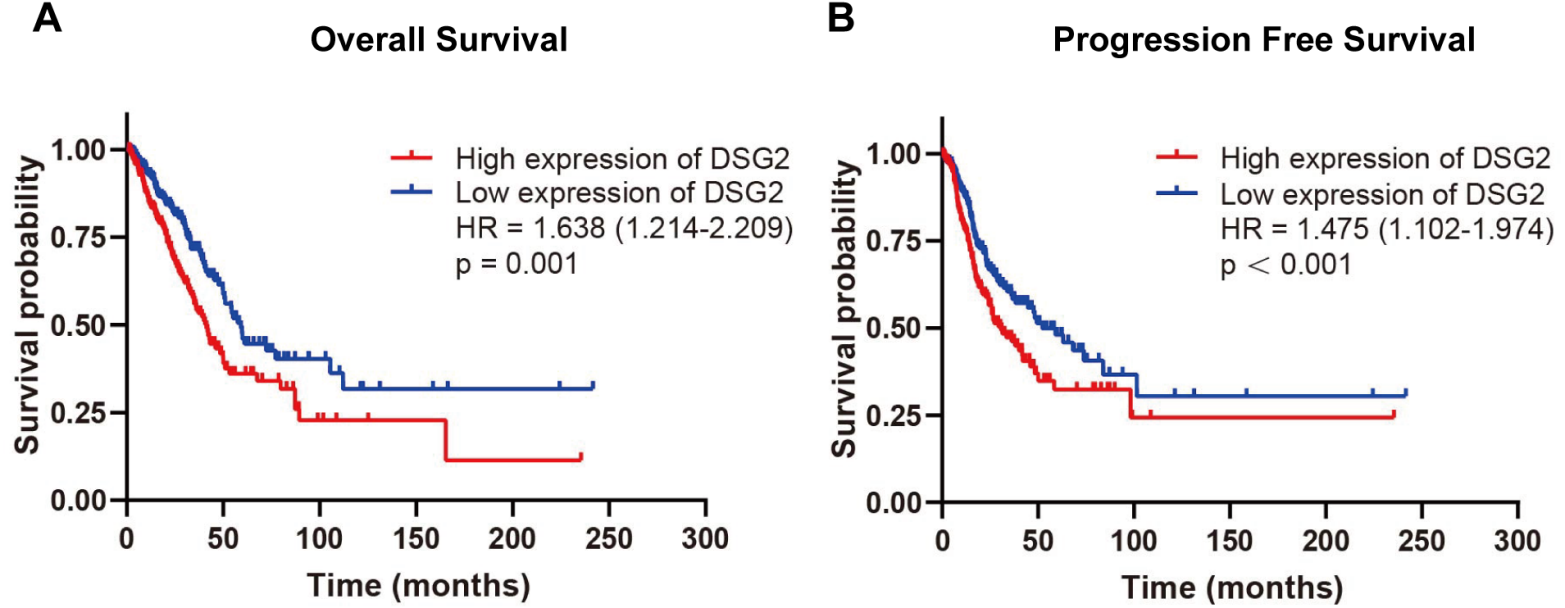
Survival analysis of DSG2 in LUAD. Patients with high DSG2 expression had a significantly short overall survival (A) and poor progression free survival (B) in TCGA.

To evaluate the relationship between DSG2 and prognosis, Kaplan–Meier survival analysis was performed. The exclusion criteria were overall survival of <30 days. The expression of DSG2 was cut into high expression level and low expression level according to the median value of DSG2. As the result shown in Fig. 4, high mRNA expression of DSG2 was associated with shorter overall survival (hazard ratio [HR]: 1.638; 95% CI [1.214–2.209], *p* = 0.001) and poor progression-free survival (HR: 1.475; 95% CI [1.102–1.974], *p* < 0.001). Furthermore, multivariate Cox regression analysis was performed to explore factors associated with survival. A trend was observed in which low DSG2 expression was associated with an increased survival time. However, no statistical significance was determined (Table 4).

**Table 4:** Univariate and multivariate analyses of overall survival in TCGA.

Parameters	Univariate analysis				Multivariate analysis			
	*p*-value	HR	95% CI		*p*-value	HR	95% CI	
Age	0.755	1.054	0.756	1.470				
Gender	0.588	0.922	0.697	1.237				
Smoking history	0.258	0.690	0.363	1.312				
TNM tumor stage	<0.001^**^	1.694	1.472	1.948	0.068	1.458	0.973	2.186
T	<0.001^**^	1.547	1.283	1.866	0.072	1.231	0.982	1.544
N	<0.001^**^	1.709	1.438	2.030	0.285	1.214	0.851	1.731
M	0.003^**^	2.257	1.316	3.869	0.621	0.775	0.282	2.128
DSG2	0.001^**^	1.638	1.214	2.209	0.461	1.151	0.792	1.673

**Notes.**

** *p* < 0.01.

T tumor N node M metastasis TNM tumor-node-metastasis HR hazard ratio CI confidence interval

### Co-expressed genes and potential pathways associated with DSG2

**Figure 5:**
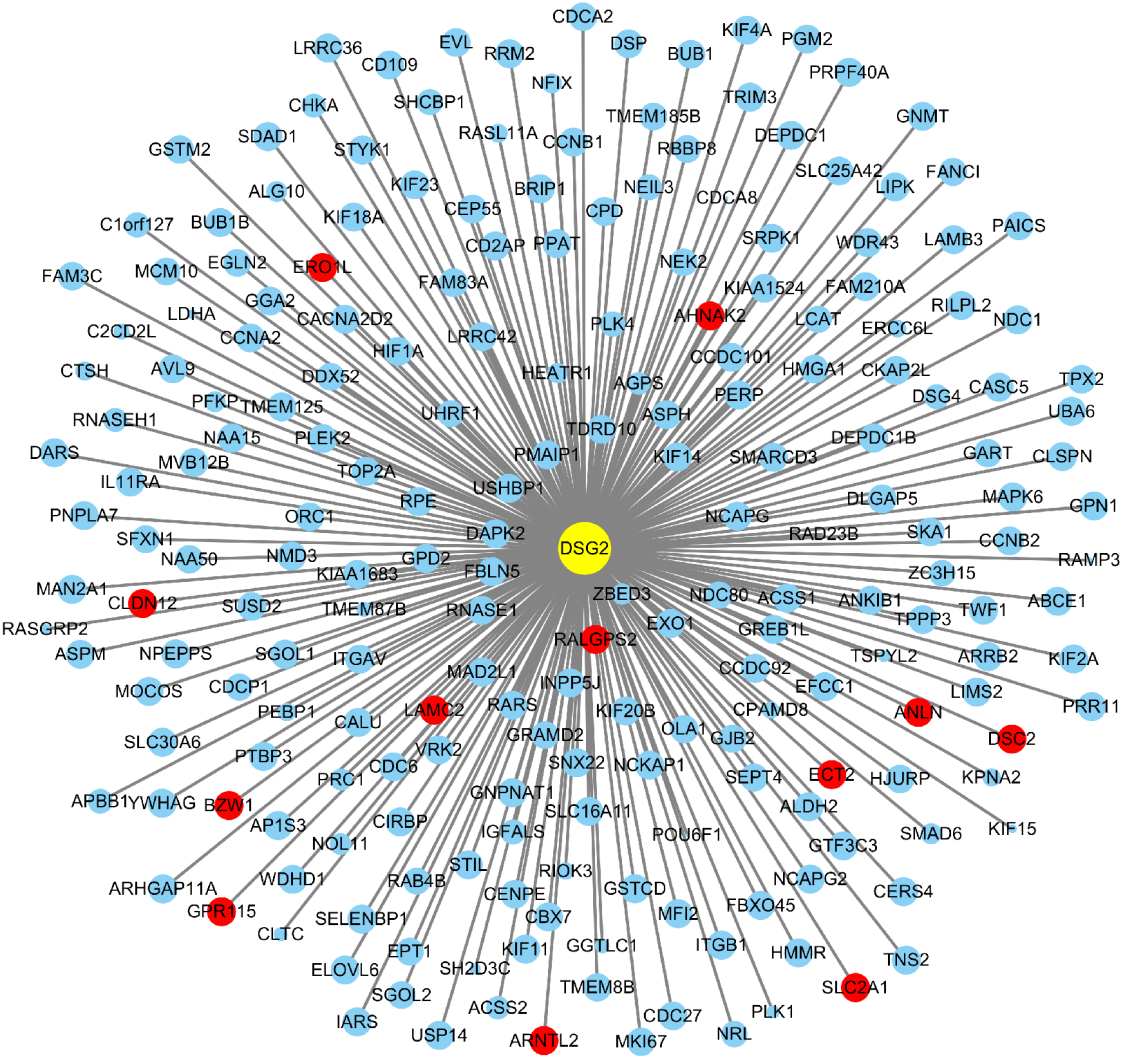
A total of 215 co-expressed genes were processed by the Cytoscape 3.7.1 software to generate a visual image based on LUAD. Blue dots represent 0.5 > |*R*| > 0.4. Red dots represent |*R*| ≥ 0.5. The size of the dots represents *p*-value. The smaller the *p*-value, the larger the dot.

**Figure 6:**
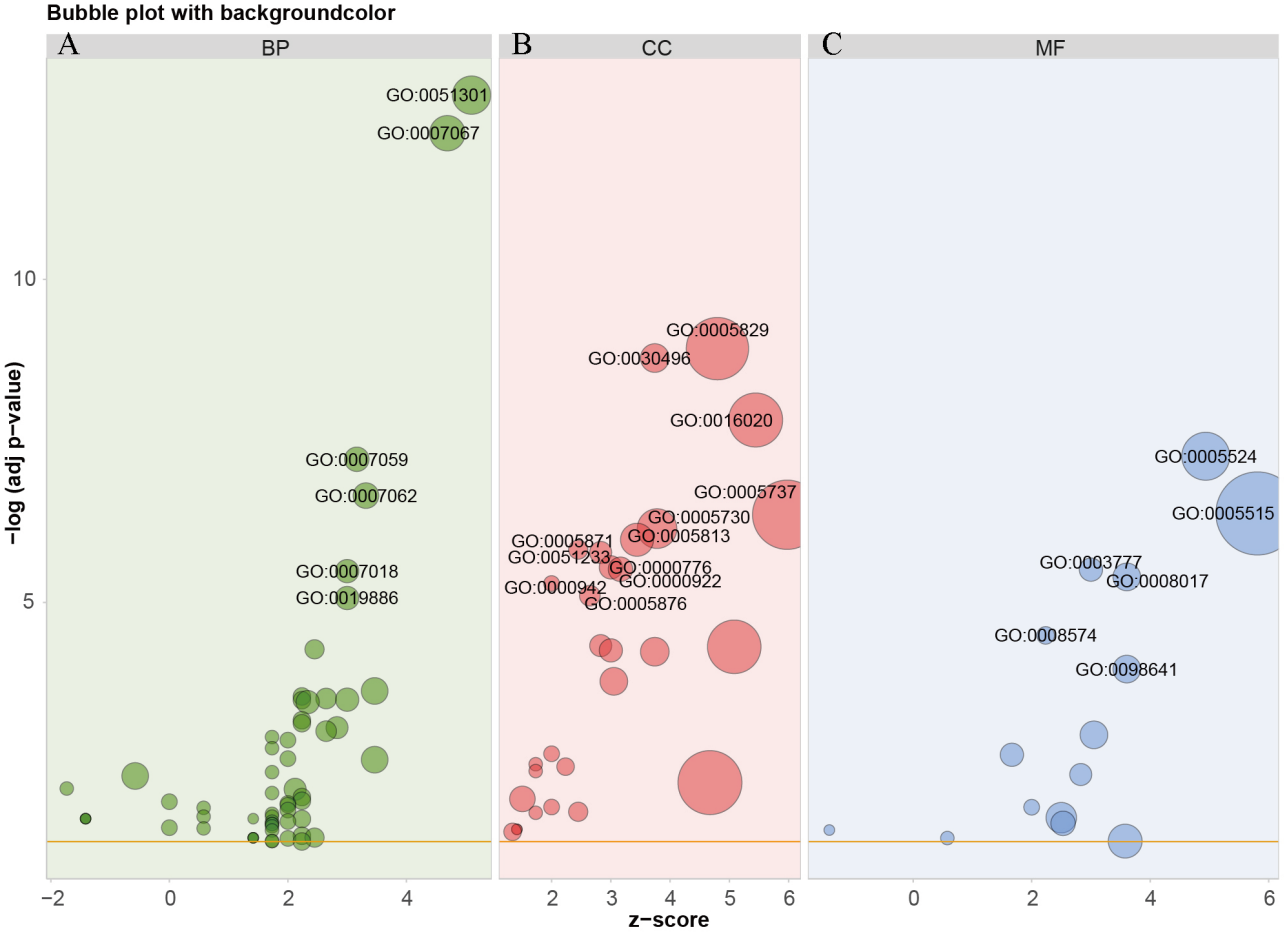
GO analysis of the co-expressed genes with DSG2 in LUAD. The bubble size represents the number of genes in one GO term. The more genes, the larger the bubble size. The horizontal axis *z*-score was calculated by the formula *z*-score = (up–down)/ √ count, up represent the number of over-expressed genes in one term, down represent the number of low-expressed genes, count means the total of genes. The GO enrichment analysis in BP were showed in green bubbles (A), CC were showed in red bubbles (B), MF were showed in blue bubbles (C). BP, Biological process; CC, Cellular Component; MF, Molecular function.

**Figure 7:**
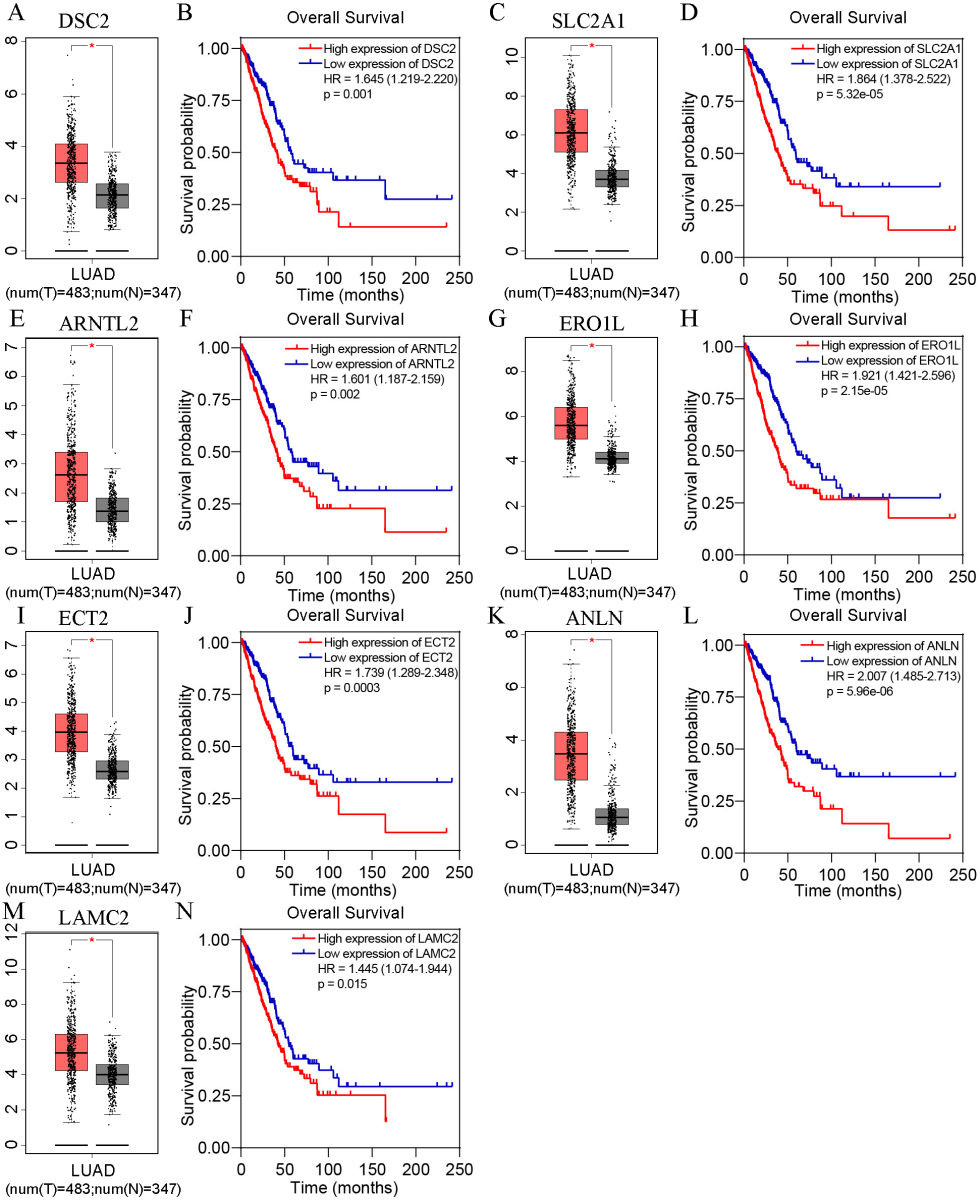
The expression and survival analysis of the co-expressed genes from GEPIA online database. In each box-and-whiskers plot, the red box represents the LUAD tissues and the grey box represents the normal lung tissues. The expression of DSC2 (A), SLC2A1 (C), ARNTL2 (E), ERO1L (G), ECT2 (I), ANLN (K), LAMC2 (M) was highly expressed in LUAD compared to normal lung tissues. High expression of DSC2 (B), SLC2A1 (D), ARNTL2 (F), ERO1L (H), ECT2 (J), ANLN (L), LAMC2 (N) had shorter overall survival. *p* < 0.05.

Based on TCGA, 215 co-expressed genes with DSG2 were identified (Fig. 5). Then GO enrichment analysis and KEGG pathway analysis were processed. As the result shown in Fig. 6 and Table 5, those co-expressed genes were mainly enriched in ‘cell division’, ‘mitotic nuclear division’, ‘chromosome segregation’ in Biological Process; ‘cytosol’, ‘midbody’ and ‘membrane’ in Cellular Component; ‘ATP binding’, ‘protein binding’, ‘microtubule motor activity’ in Molecular Function. KEGG pathway analysis revealed a significant enrichment of the co-expressed genes in the pathway ‘cell cycle’. The ten genes most strongly correlated with DSG2 are DSC2, SLC2A1, BZW1, ARNTL2, ERO1L, CLDN12, ECT2, ANLN, LAMC2 and GPR115. The expression and the correlation with OS of these ten genes were validated from GEPIA database. The results showed that seven of ten co-expressed genes were both significantly higher in LUAD compared to normal lung tissues and with worse OS (DSC2, SLC2A1, ARNTL2, ERO1L, ECT2, ANLN and LAMC2) (Fig. 7).

**Table 5:** GO enrichment and KEGG analysis of the co-expressed genes with DSG2 in LUA.

Category/term	Count	*p*-value	FDR
Biological process			
GO: 0051301 ∼cell division	26	0.074	<0.001
GO: 0007067 ∼mitotic nuclear division	22	0.063	<0.001
GO: 0007059 ∼chromosome segregation	10	0.029	<0.001
GO: 0007062 ∼sister chromatid cohesion	11	0.031	<0.001
GO: 0007018 ∼microtubule-based movement	9	0.026	0.005
GO: 0019886 ∼antigen processing and presentation of exogenous peptide antigen via MHC class II	9	0.026	0.013
Cellular component			
GO: 0005829 ∼cytosol	73	<0.001	<0.001
GO: 0030496 ∼midbody	14	<0.001	<0.001
GO: 0016020 ∼membrane	54	<0.001	<0.001
GO: 0005737 ∼cytoplasm	91	<0.001	<0.001
GO: 0005730 ∼nucleolus	28	<0.001	<0.001
GO: 0005813 ∼centrosome	19	<0.001	0.001
GO: 0051233 ∼spindle midzone	6	<0.001	0.002
GO: 0005871 ∼kinesin complex	8	<0.001	0.002
GO: 0000776 ∼kinetochore	9	<0.001	0.004
GO: 0000922 ∼spindle pole	10	<0.001	0.004
Molecular function			
GO: 0005524 ∼ATP binding	42	<0.001	<0.001
GO: 0005515 ∼protein binding	133	<0.001	<0.001
GO: 0003777 ∼microtubule motor activity	9	<0.001	0.004
GO: 0008017 ∼microtubule binding	13	<0.001	0.006
GO: 0008574 ∼ATP-dependent microtubule motor activity, plus-end-directed	5	<0.001	0.044
Pathway			
hsa04110: Cell cycle	11	<0.001	0.002

**Notes.**

FDR false discovery rate hsa Homo sapiens

In order to determine whether DSG2 plus the co-expressed genes could generate a synergistic effect for OS, all gene data were downloaded from TCGA. The expression of each gene was cut into high expression level and low expression level. Cox analyses were performed to calculate the HR. Unfortunately, HR of DSG2 +DSC2 was lower than DSG2 or DSC2 alone (1.349 *vs* 1.638 *vs* 1.645). There was no synergistic effect when DSG2 combined with the co-expressed genes. This phenomenon was also found in other six genes (SLC2A, ARTNL2, ERO1L, ECT2, ANLN and LAMC2) (Table 6).

**Table 6:** Overall survival analysis of DSG2 combined with the co-expressed genes based on TCGA.

Gene	Survival analysis			
	*p*-value	HR	95% CI	
DSG2	0.001^**^	1.638	1.214	2.209
DSC2	0.001^**^	1.645	1.219	2.22
DSG2+DSC2	<0.001^**^	1.349	1.144	1.591
SLC2A1	<0.001^**^	1.864	1.378	2.522
DSG2+SLC2A1	<0.001^**^	1.436	1.210	1.705
ARNTL2	0.002^**^	1.601	1.187	2.159
DSG2+ARNTL2	<0.001^**^	1.397	1.171	1.667
ERO1L	<0.001^**^	1.921	1.421	2.596
DSG2+ERO1L	<0.001^**^	1.501	1.255	1.795
ECT2	<0.001^**^	1.739	1.289	2.348
DSG2+ECT2	<0.001^**^	1.436	1.204	1.713
ANLN	<0.001^**^	2.007	1.485	2.713
DSG2+ANLN	<0.001^**^	1.508	1.264	1.799
LAMC2	0.015^*^	1.445	1.074	1.944
DSG2+LAMC2	<0.001^**^	1.337	1.125	1.590

**Notes.**

* *p* < 0.05.

** *p* < 0.01.

HR hazard ratio CI confidence interval

## Discussion

In this study, we have investigated the clinical value of DSG2 in lung adenocarcinoma. Quantitative RT-PCR data, TCGA data and Oncomine datasets were utilized to validate the high expression of DSG2 in lung adenocarcinoma. The expression of DSG2 protein was also higher in lung adenocarcinoma by tissue microarray and HPA database. In order to explore the role of DSG2 in the development of lung adenocarcinoma, the relationship between DSG2 and the clinicopathological parameters were analyzed. We found that high DSG2 expression was positively correlated with TNM stage, tumor size, lymph node metastasis and poor prognosis of LUAD patients. Pearson analysis was used to detect co-expressed genes with DSG2 in LUAD. Seven of top ten genes showed high expression in LUAD according to GEPIA database. High expression of these genes had a shorter overall survival time.

Multiple studies have shown that DSG2 is deregulated in many types of human tumors. Because of the heterogeneity of cancers, its expression trends are not the same in different tumors. For example, DSG2 is over-expressed in malignant skin carcinomas (Brennan & Mahoney, 2009) and low-expressed in pancreatic cancer (Ramani, Hennings & Haun, 2008). Saaber et al. (2015) detected DSG2 protein expression in 112 primary lung cancer samples (including 82 LUSC cases and 22 LUAD cases) by IHC and found that the expression of DSG2 was significantly higher in LUSC than in LUAD (*p* = 0.009). Cai et al. (2017) detected the DSG2 expression in 28 paired NSCLC and normal tissue samples by qRT-PCR and in 70 cases of paraffin-embedded NSCLC tissues. The results demonstrated that both DSG2 mRNA and protein were highly expressed in NSCLC. These findings were consistent with our study. Interestingly, our previous studies also demonstrated that DSG2 was over-expressed in NSCLC. In 2014, we investigated a differential expression profile between lung adenocarcinoma and normal lung tissue by iTRAQ labeling combined with 2D-LC-MS/MS. Bioinformatic analysis showed that DSG2 was 1.5-fold higher in lung adenocarcinoma than normal lung tissue (Zhang et al., 2014). In 2016, we investigated differences in the mitochondrial protein profiles between lung adenocarcinomas and normal tissue. Differentially expressed proteins were identified and DSG2 was one of them (Li et al., 2017).

It has been reported that desmosomal cadherin related genes play an important role in the proliferation and metastasis of many human cancers in recent years (Ormanns et al., 2015; Wang et al., 2016; Yang et al., 2015). DSG2 is also involved in the occurrence, development and metastasis of tumors. However, the roles in different tumors are not the same. DSG2 present in breast cancer cells may function as a tumor suppressor molecule (Davies et al., 1997). In pancreatic adenocarcinoma cells, silencing of DSG2 resulted in loss of cell cohesion and improved migration and invasion (Hutz et al., 2017). However, in colon cancers and NSCLC, knockdown DSG2 suppressed cell proliferation both in vitro and in vivo. DSG2 functions through many ways. Downregulation of DSG2 inhibited cell proliferation through altered phosphorylation of epidermal growth factor receptor (EGFR) and downstream extracellular signal-regulated kinase activation (Kamekura et al., 2014). Further study shows that overexpression of DSG2 in human skin squamous cell carcinoma cell line enhances EGFR activation and increases cell proliferation and migration through a c-Src and EGFR dependent manner (Overmiller et al., 2016). DSG2 knockdown also downregulated cyclin-dependent kinase 2 expression and upregulated p27 expression in NSCLC (Cai et al., 2017). A recent study showed that overexpression of DSG2 induced STAT3 phosphorylation and further increased Gli1 levels in basal cell carcinomas (Brennan-Crispi et al., 2019).

DSG2 can be a prognostic marker in cancers. Reduced level of DSG2 was a significant independent marker of poor clinical outcome in prostate cancer (Barber et al., 2014). On the contrary, increased DSG2 expression may function as a promising marker for unfavorable prognosis of hepatocellular carcinoma (Han et al., 2018). In our study, DSG2 might be an independent risk factor for lung adenocarcinoma.

DSG2 is closely related to DSC2. Lie, Cheng & Mruk (2010) proposed a novel regulatory protein complex composed DSG2, DSC2, c-Src, coxsackie and adenovirus receptor and ZO-1 at the blood-testis barrier. Another study showed that DSC2 was compensatory increased in DSG2-deficient cells, while downregulated DSC2 restored proliferation in DSG2-deficient cells (Kamekura et al., 2014). Besides DSC2, we found six genes (SLC2A, ARTNL2, ERO1L, ECT2, ANLN, LAMC2) were co-expressed with DSG2 in LUAD. Solute carrier family 2 (SLC2A) is a uniporter protein encoded by the SLC2A1 gene and functions as a pivotal rate-limiting element in the transport of glucose in tumor cells (Zhang, Xie & Li, 2019). ARNTL2 is a paralog of the circadian transcription factor ARNTLl and drives metastatic self-sufficiency by orchestrating the expression of a complex pro-metastatic secretome in lung adenocarcinoma (Brady et al., 2016). Endoplasmic reticulum oxidoreductase 1 alpha (ERO1L) is an endoplasmic reticulum (ER) luminal glycoprotein and favors disulfide bond formation by selectively oxidizing PDI (Cabibbo et al., 2000). Epithelial cell transforming sequence 2 (ECT2) is a guanine nucleotide exchange factor (GEF) of the Rho GTPase family. It regulates cell division and cell cycle (Wang et al., 2018). Anillin (ANLN) encodes an actin-binding protein that consists of 1125 amino acids and plays an important role in cytokinesis (Wang et al., 2019). Laminin γ2 (LAMC2), LAMB3 and LAMA3 constitutes the heterotrimeric glycoprotein laminin-332, then regulate the migration of epithelial cells and promote cancer invasion (Masuda et al., 2012). To date, no studies on these genes and DSG2 could be found.

In order to explore the potential mechanism of the 215 co-expressed genes, GO and KEGG enrichment analysis were performed. According to GO enrichment, the co-expressed genes correlated with DSG2 might have an important effect on the progression of LUAD by modulating several cellular biology processes, such as cell division. Moreover, these genes might be closely related with cell cycle related pathway. More studies are needed in the future.

## Conclusion

All in all, our data showed that the mRNA and protein levels of DSG2 were enhanced in LUAD. High expression of DSG2 was positively associated with TNM stage, tumor size and lymph node metastasis. LUAD patients with high DSG2 expression had shorter overall survival and poor progression-free survival. Co-expressed genes correlated with DSG2 were mainly enriched in ‘cell division’, ‘mitotic nuclear division’, ‘chromosome segregation’ in BP; ‘cytosol’, ‘midbody’ and ‘membrane’ in CC; ‘ATP binding’, ‘protein binding’, ‘microtubule motor activity’ in MF; ‘cell cycle’ in KEGG pathway analysis. Further studies are still needed to investigate the potential mechanism of DSG2 in LUAD.

##  Supplemental Information

10.7717/peerj.8420/supp-1 Supplemental Information 1 Clinicopathological features downloaded from TCGA were analyzed

10.7717/peerj.8420/supp-2 Supplemental Information 2 Raw data: The expression of DSG2 between lung adenocarcinoma and normal lung tissues based on TCGA

10.7717/peerj.8420/supp-3 Supplemental Information 3 Raw data for qRT-PCR

## Additional Information and Declarations

### Data Availability

The following information was supplied regarding data availability: The raw measurements are available in the Supplementary Files.
